# A compound combination screening approach with potential to identify new treatment options for paediatric acute myeloid leukaemia

**DOI:** 10.1038/s41598-020-75453-3

**Published:** 2020-10-28

**Authors:** Katrina M. Lappin, Lindsay Davis, Kyle B. Matchett, Yubin Ge, Ken I. Mills, Jaine K. Blayney

**Affiliations:** 1 Patrick G Johnston Centre for Cancer Research, Queen’s University Belfast, 97 Lisburn Road, Belfast, BT9 7AE UK; 2 Northern Ireland Centre for Stratified Medicine, School of Biomedical Sciences, Ulster University, C-TRIC, Altnagelvin Hospital Campus, Derry/Londonderry, BT47 6SB UK; 3 School of Medicine, Wayne State University, 421 E. Canfield Street, Suite 3128, Detroit, MI 48201 USA

**Keywords:** Haematological cancer, Cell biology

## Abstract

Paediatric acute myeloid leukaemia (AML) is a heterogeneous disease characterised by genetics and morphology. The introduction of intensive chemotherapy treatments together with patient stratification and supportive therapy has resulted in a moderate improvement in patient prognosis. However, overall survival rates remain unacceptably poor, with only 65% of patients surviving longer than 5 years. Recently age-specific differences in AML have been identified, highlighting the need for tailored treatments for paediatric patients. Combination therapies have the potential to improve patient prognosis, while minimising harmful side-effects. In the laboratory setting, identifying key combinations from large drug libraries can be resource-intensive, prohibiting discovery and translation into the clinic. To minimise redundancy and maximise discovery, we undertook a multiplex screen of 80 apoptotic-inducing agents in paediatric AML pre-clinical models. The screen was designed using an all-pairs testing algorithm, which ensured that all pairs of compounds could be tested, while minimising the number of wells used. We identified a combination of ABT-737, a Bcl-2 family inhibitor and Purvalanol A, a CDK inhibitor, as a potential targeted therapy for AML patients with an MLL rearrangement and an FLT3-ITD. Our approach has the potential to reduce resource-intensity and time associated with the identification of novel combination therapies.

## Introduction

Acute leukaemia is the most prevalent cancer diagnosed in children, adolescents and young adults (AYA), accounting for approximately 30% of cancer cases. While, acute myeloid leukaemia (AML) makes up only one fifth of all acute leukaemia cases, it accounts for more deaths than the more common acute lymphocytic leukaemia (ALL). This can be attributed to the relatively higher risk of relapse among AML patients, with a corresponding five-year overall survival (OS) rate of 60 to 70% in comparison to 90% in ALL^1,2^. Patients with refractory disease have a significantly worse outcome, with the five-year OS rate reducing to 30% for those experiencing early relapses^3^. To date, our understanding of paediatric AML has been inferred from genomic data collated from adult cases. With the high-throughput sequencing of paediatric samples, it has become apparent that the mutational landscape of paediatric AML differs widely from adult AML, with age-specific alterations more evident^4,5^. Mutations in genes such as NRAS, KIT and KRAS are more common in paediatric AML, whilst mutations in DNMT3A and NPM1 are associated with adult cases^5^. Currently, there are no targeted therapies for paediatric AML, with the standard treatment consisting of Cytarabine plus an anthracycline agent. This has remained largely unchanged for four decades^6^, with the Food and Drug Administration (FDA)-approval of Gemtuzumab ozogamicin being one of the few breakthroughs for the treatment of patients with relapse/refractory CD33^+^ AML^3,5,7^. The significant side-effects of these therapies can affect survivors for the rest of their lives, with cardiac dysfunction and later health complications such as subsequent malignancies^8^. In addition, 10–20% of children and young people do not achieve remission during induction therapy, therefore the identification of novel targeted therapies has the potential to improve treatment outcomes while reducing both side-effects and the risk of refractory disease^9–11^. Combination treatments are preferable to monotherapy due to their superior efficacy at targeting tumour growth in an additive or synergistic manner. Combinations of therapies also allow lower doses to be used, therefore reducing potential harmful acute and chronic side effects, whilst also reducing the potential for drug resistance and relapse^11,12^. However, the identification of effective combination therapies has its challenges, including the complex screening process, identification of synergistic dose ranges and whether therapies are given in a sequential or simultaneous order for optimal response. These limitations are common to many disease contexts; Tan et al. was the first to propose a multiplex screening approach in order to identify new compound combinations derived from large reference libraries, for the treatment of the human immunodeficiency virus (HIV)^13^.

In this paper we show how an alternative multiplex screening approach, using a design adapted from error-testing in computer science^14^, can facilitate compound combination testing in paediatric AML. Using a reference library of 80 apoptotic-inducing agents, this novel approach maximised the probability of identifying new compound pairs with therapeutic potential, while minimising replication and redundancy. Thus, we identified the combination of ABT-737, a B-cell lymphoma (BCL)-family inhibitor, and Purvalanol A, a cyclin dependent kinase (CDK)-inhibitor, as a potential targeted therapy for AML patients carrying a mixed lineage leukaemia (MLL) rearrangement and internal tandem duplications of the fms-related tyrosine kinase 3 gene (FLT3-ITD).

## Results

### In silico analysis of RNA sequencing data identified apoptosis as a targetable pathway in paediatric AML patients

**Figure 1:**
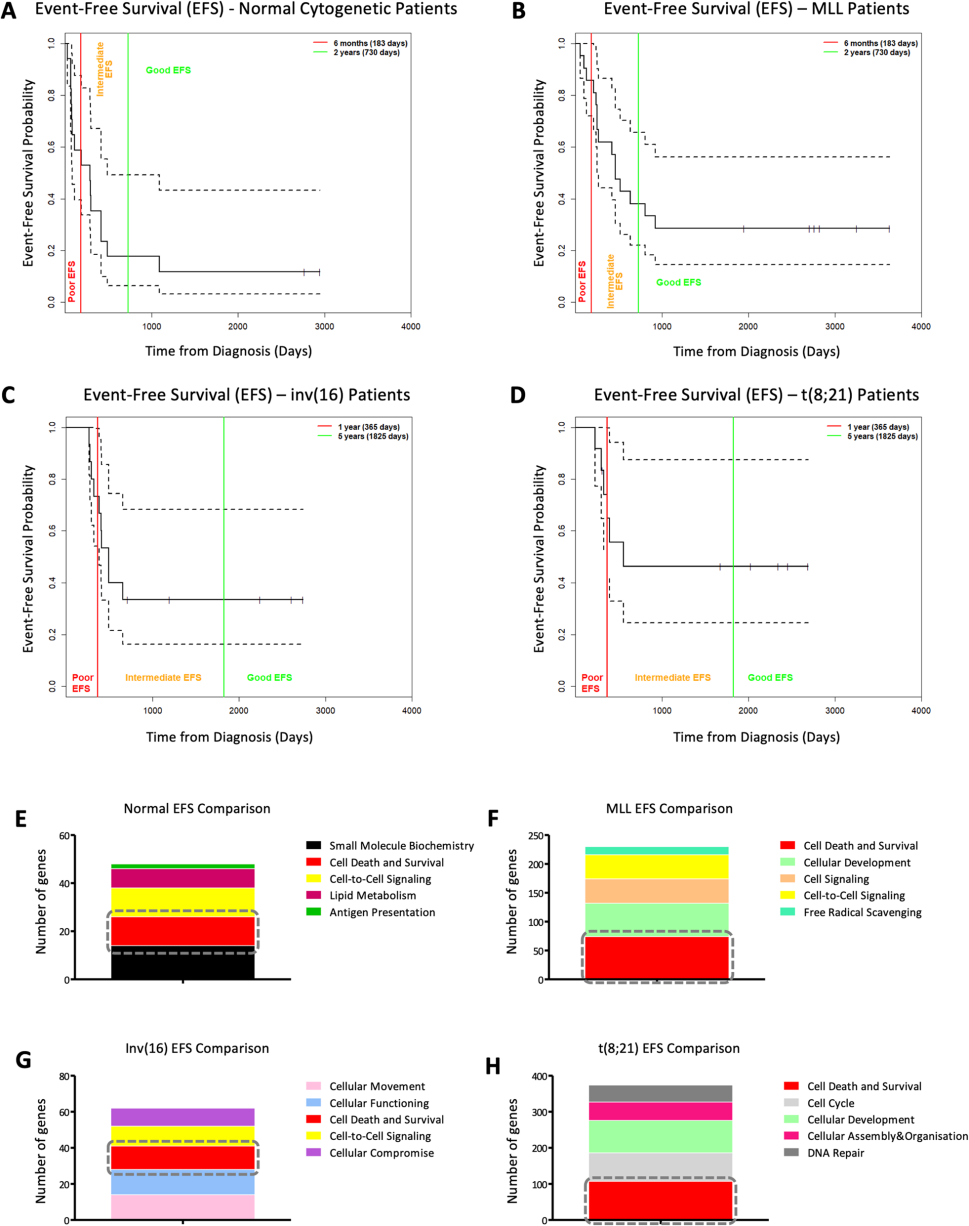
Identifying a therapeutic target in paediatric AML. Kaplan–Meier curves, generated for each cytogenetic risk group, (**A**) normal cytogenetic, (**B**) MLL, (**C**) inv(16) and (**D**) t(8;21), and, showing good (green), intermediate (orange) and poor (red) prognosis using event-free survival (EFS). Good prognosis was defined as an EFS of greater than 730 days (normal cytogenetics or MLL rearrangement) or 1825 days (inv(16) or t(8;21) translocation), bad prognosis was defined as an EFS of less than 183 days (normal cytogenetics or MLL rearrangement) or 365 days (inv(16) or t(8;21) translocation). Dysregulated pathways within each cytogenetic group, derived from IPA analysis of prognosis-associated DEGs (**E**) normal cytogenetic, (**F**) MLL, (**G**) inv(16) and (**H**) t(8;21). Cell death and survival (red) was common to all four groups. The x-axis (molecules) denotes the number of genes differentially expressed within each risk group and involved in the pathways determined with IPA.

Clinical data corresponding to paediatric AML cases from the TARGET AML initiative were extracted (https://ocg.cancer.gov/programs/target/data-matrix). Analysis of EFS, using Kaplan–Meier estimation and clinically-relevant thresholds, was used to identify good and poor prognostic patients within each of four cytogenetic risk groups, normal cytogenetics, MLL, inv(16) and t(8;21) (Fig. 1A–D). Prior to DEG analysis, within each four cytogenetic risk group, good and poor prognostic patients were balanced for confounding factors. This resulted in three patients per prognostic group (balanced and unbalanced summaries for each cytogenetic group are available in Fig. S1). Next, within each cytogenetic group, patients with poor prognosis were compared to those with good prognosis to generate lists of DEGs (Supplementary Tables 2–5), which were in turn analysed to identify aberrant targetable pathways. This identified cell death and survival as a common dysregulated and therapeutically targetable pathway (Fig. 1E–H) with p-values ranging from 1.38e^−4^–4.9e^−2^ (normal cytogenetics), 9.38e^−8^–1.12e^−2^ (MLL), 1.43e^−7^–4.34e^−2^ (inv(16)) and 3.35e^−19^–1.66e^−3^ (t(8;21)).

### Temporal multi-cell line single-agent screen established common dose concentration across 80 apoptosis-inducing agents

**Figure 2:**
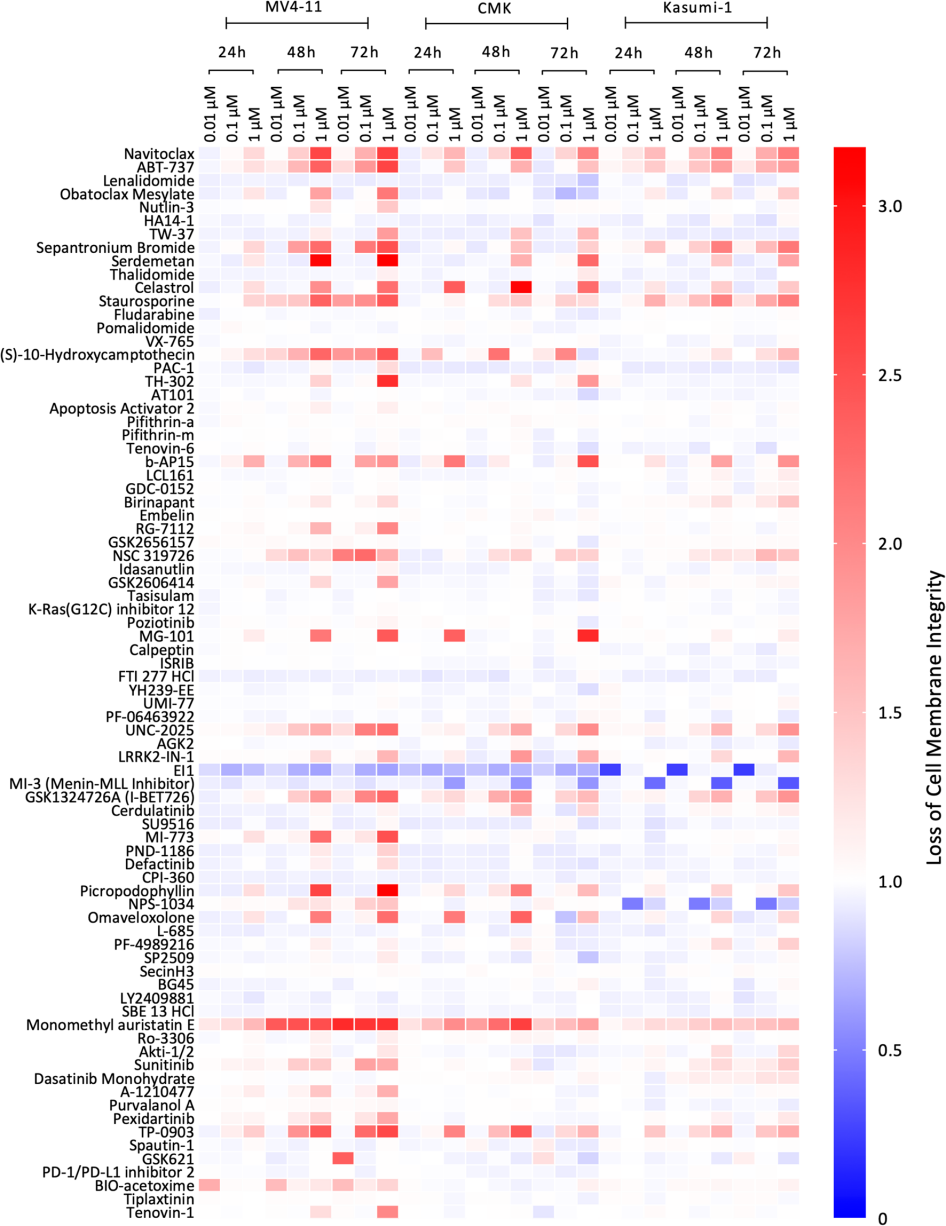
Targeting paediatric leukaemia cells with apoptosis inducing agents. A heatmap summarising the viability/proliferation response of each cell line, MV4-11, CMK and Kasumi-1, to the 80 compounds. Red shows an increased relative fluorescence unit (RFU) compared to the vehicle and therefore cell death, while blue indicates the cells are alive and actively proliferating.

To validate apoptotic processes as targetable pathways in paediatric AML and select a dose for combination screening, we considered 80 apoptosis-inducing compounds (Selleckchem, UK) in three paediatric AML cell lines (MV4-11, CMK and Kasumi-1) at three concentrations (0.01, 0.1 and 1 µM) and assessed cell viability at several time-points up to 72 h. Fluorescent readings for each compound were normalised to a DMSO vehicle control and any compound with an RFU value greater than or equal to 2 indicating that cell death had occurred and was considered a successful treatment with anti-leukaemic effects (Fig. 2, normalised data Supplementary Table 6). Due to the mutational and cytogenetic diversities of these cell lines different responses to each compound were observed, with certain compounds showing induced cell death specificity for the MV4-11 cell line (b-AP15, UNC-2025 and Sunitinib) and only Monomethyl Auristatin E, an antineoplastic agent that disrupts microtubule formation, induced cell death in all three cell lines at each concentration at all time points (Supplementary Fig. S2). Interestingly UNC-2025, a dual MER/FLT3 inhibitor, was specific to MV4-11 cells, the only cell line in the panel tested to carry a FLT-ITD. From the single agent screen, we selected 0.1 µM as the concentration to be used for each compound in the combination approach. This allowed for the minimisation of cytotoxicity for the maximum number of compounds across the three cell lines.

### Redundancy minimised and discovery maximised using multiplex screening based on an all-pairs testing algorithm

**Figure 3:**
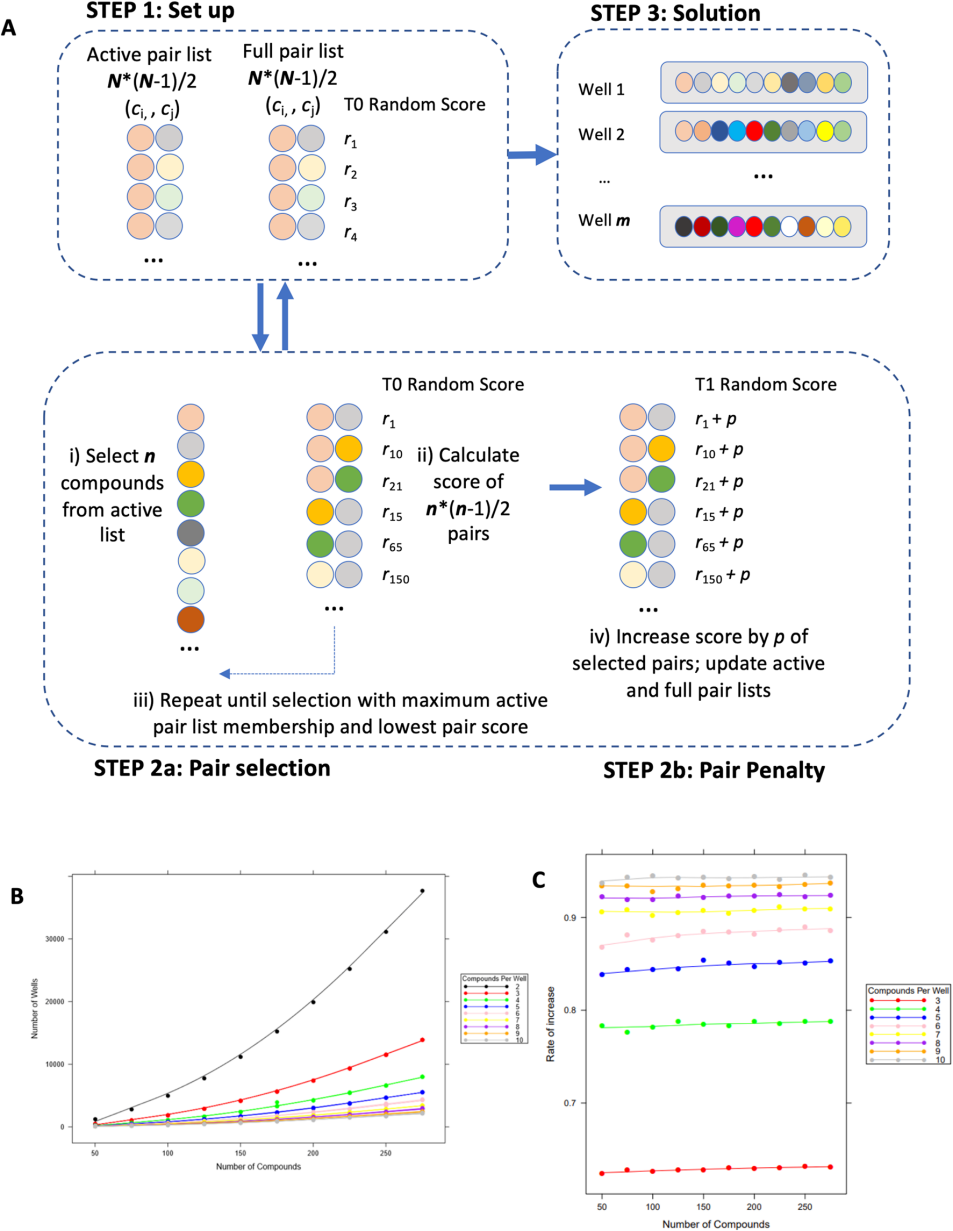
Development of the algorithm. (**A**) Schematic of the all-pairs testing algorithm, a technique commonly used in computing science to identify a combination of bugs/errors in software development, was adapted to identify compound combinations. (**B**) All-pairs testing algorithm calibration showing that with an increasing number of compounds the well usage increases, however when more compounds are added to a well the well usage is reduced. (**C**) Plot of rate of increase (decrease) (median over five iterations) in number of wells, when adding additional compounds to each well, by number of compounds per well and total number of compounds.

Using standard approaches, to test all possible pair-wise combinations for 80 apoptosis-inducing compounds from the initial screen would require 3,160 individual wells (= 80 × (80 – 1)/2). Using our all-pairs testing algorithm (50 repeated iterations), summarised in Fig. 3A, a reference library of this size (*N* = 80) could be accommodated by using ten (*n*) compounds per well over 160 wells (*m*), with 45 possible pairs in each of the 160 wells. Calibration analysis indicated as expected, that with each additional compound, i.e. an increase in *n*, added to a well, the number of wells required decreased, i.e. *m* reduced (Fig. 3B), with no significant reduction in well usage being observed between using eight or ten compounds per well. As the size of the reference library increased (*N*), the advantages of using an increased number of compounds could be observed. Though the rate of increase at which this benefit is accrued begins to slow after n = 3 (Fig. 3C).

**Figure 4:**
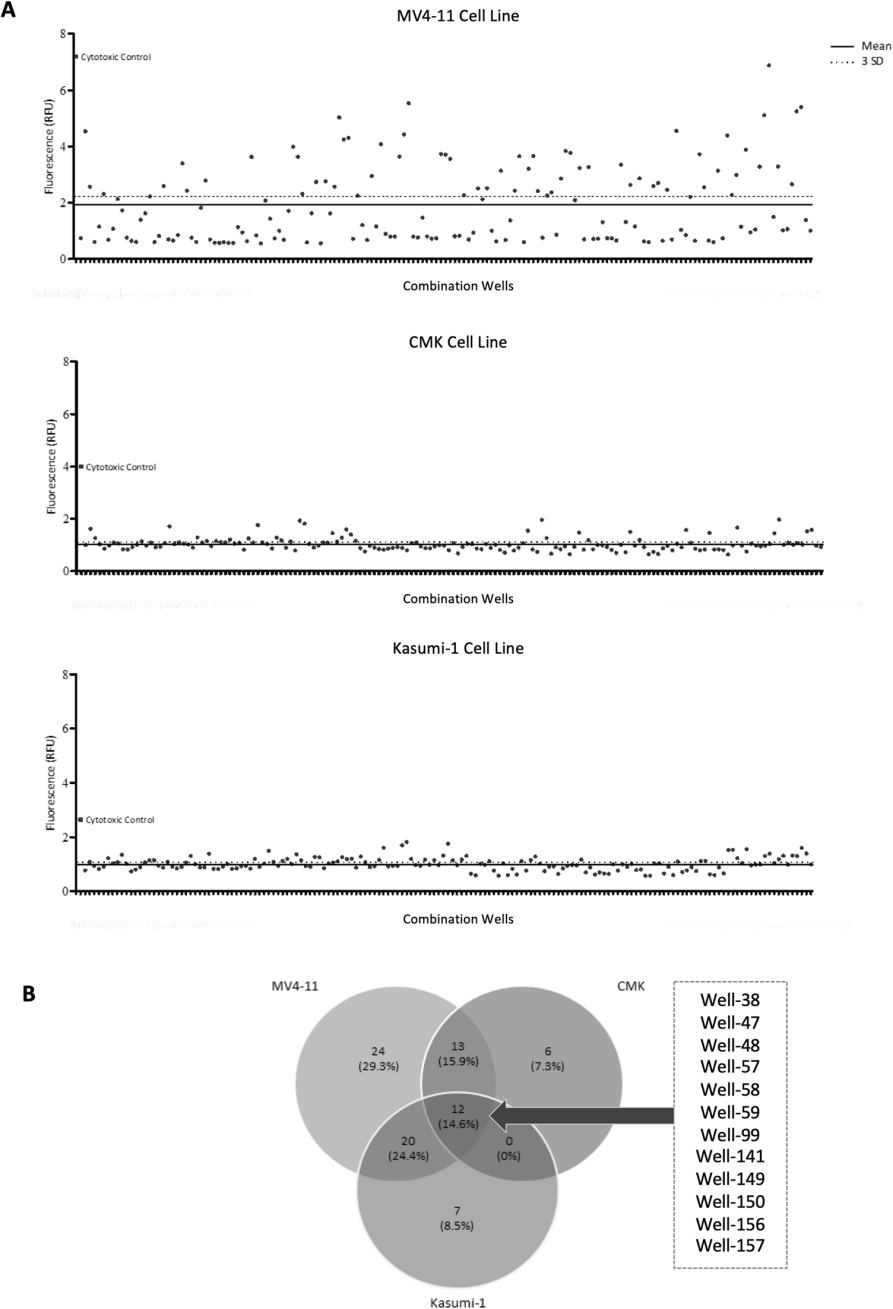
Algorithm generated combination screen against paediatric AML cell lines. The response of each cell line (**A**) MV4-11, CMK and Kasumi-1, at the 72-h time point to each of the 160 combination wells (x-axis). The mean (solid line) and three standard deviations (3SD) from the mean (dotted line) were calculated for all data points to identify successful wells (RFU values greater than 3SD). (**B**) Venn diagram summarising the successful wells (wells which induces a response of RFU values greater than 3SD from the mean) which were common and unique across the cell lines at the 72-h time point.

Using the optimum dose of 0.1 µM, three cell lines were each treated with different combinations of ten of the 80 compounds across 160 wells and assessed at 24 h, 48 h and 72 h. In 82 wells, cell viability was reduced across the panel of cell lines at 72 h (Fig. 4A). Cell viability data for all wells at the three time points is available in Supplementary Fig. S3. Similarly, to the single agent screen, each cell line behaved differently to each of the combination wells. In a total of 12 wells (out of 160) a common effect was observed, with an increase in RFU being observed indicating a degree of cell death was achieved. In contrast, a total of 37 wells showed specificity to one of the three cell lines (Fig. 4B). Perhaps surprisingly, 48.75% (78 of 160) of compound combination wells had little effect on the viability of any of the cell lines.

### Deconvolution of combination wells to identify driving synergistic effects common to multiple paediatric AML cell lines

**Figure 5:**
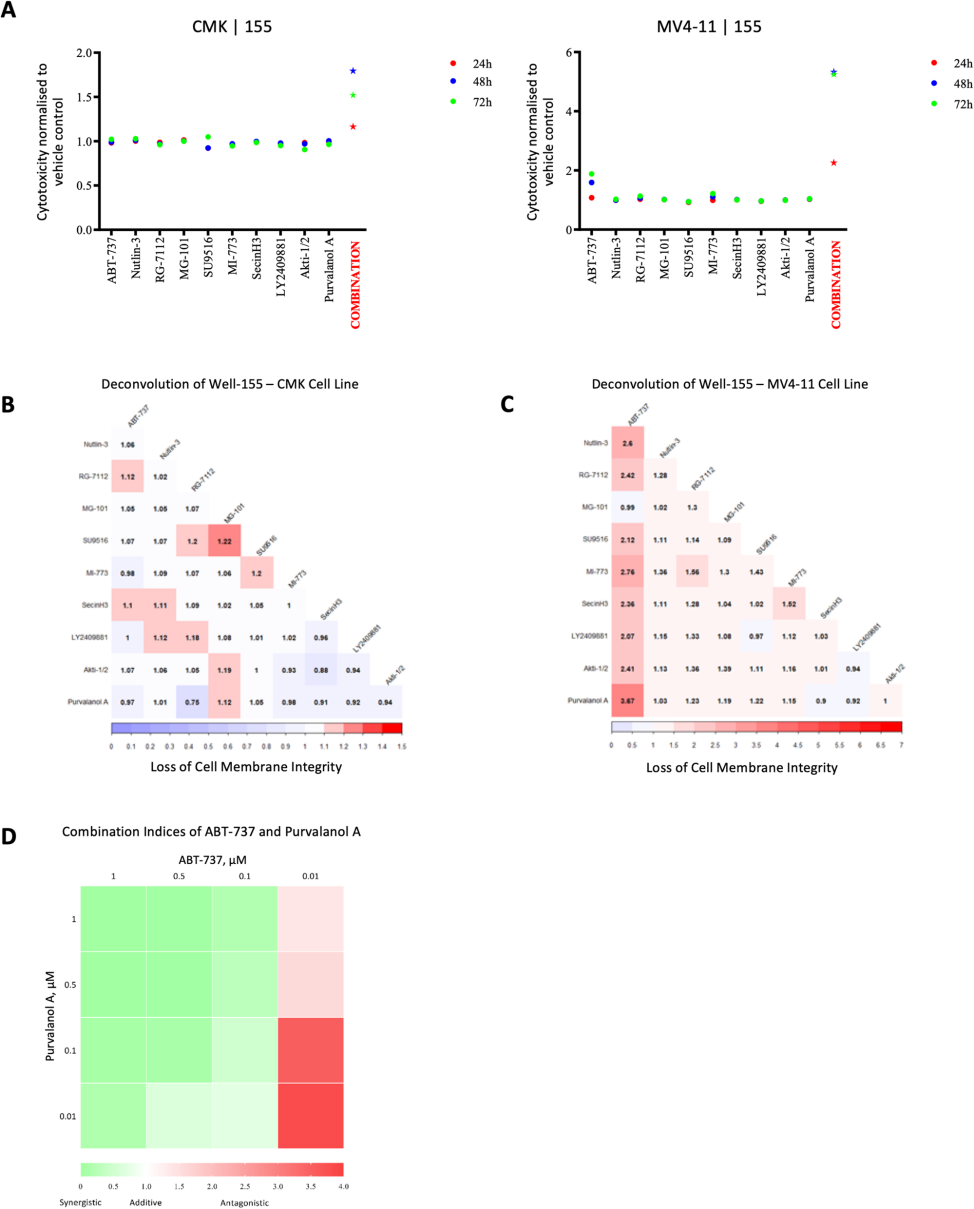
Identifying successful wells containing a potential synergistic pairing. (**A**) Response of the MV4-11 and CMK cell lines to compounds in well-155 as single agents and in the 10-drug combination at 24, 48 and 72-h. (**B**) Correlogram presenting the response of the CMK cell line to the 45 pairwise combinations present in well-155 at 72 h. Scale bar ranges from zero up to the RFU value (1.5) achieved by a cytotoxic control for the CMK cell line. (**C**) Correlogram presenting the response of the MV4-11 cell line to the 45 pairwise combinations present in well-155 at 72 h. Scale bar ranges from zero up to the RFU value (7) achieved by a cytotoxic control for the MV4-11 cell line. (**D**) Heat map showing the interaction of ABT-737 and Purvalanol A across multiple doses against the MV4-11 cell line at 72 h.

To identify wells with potential successful combinations, the increase in fluorescence were examined further. Of particular interest was replicated activity across two or more cell lines. The combined analysis of single versus combination agent/s RFUs indicated that many of the successful combination wells were due to the cytotoxic activity of a single agent alone or several agents producing an additive effect rather than a synergistic interaction, for example in well-99 (Fig. S4A) in the MV4-11 cell line the effect in combination well-99 could be attributed to an additive effect between ABT-737, Staurosporine, (S)-10-Hydrixycamptothecin and GSK1324726A, while the effect observed in the Kasumi-1 cell line from combination well-99 is mostly due to (S)-10-Hydroxycamptothecin, which was toxic for this cell line as a single agent as observed in the single agent screen of the 80 compounds, and the response observed in the CMK cell line can be linked to the toxicity of three compounds, ABT-737, Staurosporine and GSK1324726A. Only one well (well 155) contained a combination that suggested the presence of a synergistic effect in two of the cell lines (CMK and MV4-11). Each of the ten compounds (ABT-737, Nutlin-3, RG-7112, MG-101, SU9516, MI-773, SecinH3, LY2409881, Akti-1/2 and Purvalanol A) in this well had minimal or no effect as single agents on the viability of either cell line, however, a combination of two or more of the compounds resulted in a substantial increase in RFU value indicative of cell death (Fig. 5A). To identify a possible synergistic pair the ten compounds were deconvolved, resulting in 45 pairwise combinations used at 0.1 µM against the MV4-11 and CMK cells. The output for the CMK cell line was ambiguous, as several pairings were possibly identified (Fig. 5B). However, the pairing with the largest RFU value (RFU 1.22), when normalised to the vehicle control, was a combination of MG-101, a cysteine protease inhibitor, and SU-9516, a CDK inhibitor. Unlike the output from the CMK cell line, the MV4-11 data showed a key pairing that produced a substantial increase in RFU value (RFU 3.67), a combination of ABT-737, a Bcl-2 family inhibitor, and Purvalanol A, a CDK inhibitor (Fig. 5C). All time points of the deconvolution of Well-155 are available in Fig. S4B. The combination of ABT-737 and Purvalanol A showed a synergistic anti-leukaemic effect at multiple dose combinations ranging from 0.5 to 0.001 µM, as calculated using the CompuSyn software (https://www.combosyn.com), against the MV4-11 cell line up to 72 h (Fig. 5D). Interestingly, the combination of ABT-737 and Purvalanol A also appeared in another well considered successful for the MV4-11 cell line, well-6, however alternative compounds in this well appeared to produce an additive response rather than the desired synergistic response (Fig. S4C) potentially masking the synergistic interaction between ABT-737 and Purvalanol A.

### A combination of ABT-737 and Purvalanol A specifically reduces viability of AML subtypes harbouring a FLT3-ITD and an MLL rearrangement

**Figure 6:**
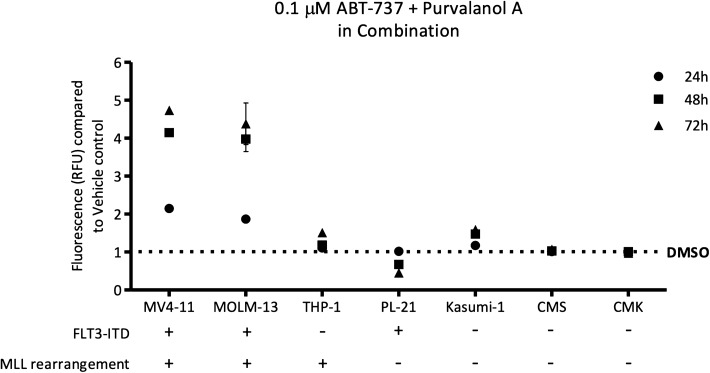
Combining ABT-737 and Purvalanol A shows specificity for cells carrying a FLT3-ITD and an MLL rearrangement. Paediatric AML cell lines, with (X) or without (–) FLT3 mutations or MLL translocations treated with ABT-737 and Purvalanol A mount substantially different responses to the combination dependent on mutation present.

Following on from successful identification of a novel compound pairing, the combination of ABT-737 and Purvalanol A was screened against a wider panel of paediatric AML cell lines (MV4-11, MOLM-13, THP-1, PL-21, Kasumi-1, CMS and CMK cell lines), to establish whether this combination specifically targeted the MV4-11 cell line or if it had the potential to reduce cell viability in a range of AML subtypes. Interestingly, the data showed that this combination specifically targets cell lines with a FLT3-ITD and an MLL rearrangement including the MV4-11 and MOLM-13 lines, whilst not impacting the viability of the THP-1 cell line, included as a control for the MLL rearrangement or the PL-21 cell line, included to control for the presence of a FLT3-ITD, or any of the other lines used (Fig. 6).

## Discussion

The treatment for AML has not changed for decades, cytarabine in combination with anthracyclines^6,15^. Since 2017 the FDA have approved seven (Midostaurin, Gilteritinib, Glasdegib, Mylotarg, Venetoclax, ivosidenib and CPX-351) new drugs for treating adult AML, with only one (Mylotarg) for paediatric AML^16–18^. In general, the discovery of new treatments for different diseases are financially restrictive, with a high risk of failure—only one in ten compound candidates may reach the clinical testing stage^19^. The lack of characterisation of disease heterogeneity, such as in paediatric AML can add further obstacles. Four decades ago approximately 30% of children diagnosed with AML survived five years or more^20^. As a result of improved diagnostic approaches, better risk stratification and more intensive chemotherapy, more patients now achieve complete remission and EFS, with approximately 65% of cases surviving five years or more^21,22^. This can come at a considerable cost, the intensification of the chemotherapy regime can leave some paediatric patients with life-long complications or result in death^23,24^. In addition, around 30% of patients relapse and one fifth of cases do not show any benefit from current treatments^25^.

Drug repositioning of approved compounds for use in different disease settings can contribute to accelerating the discovery process^26^. This may involve a combination of in silico, in vivo and/or in vitro techniques. High-throughput screening of drug libraries, in particular combination screens, have revealed novel drug pairs of therapeutic potential with respect to AML. For example, recent studies have identified synergistic effects of ruxolitinib, a JAK1/2 inhibitor, with venetoclax, a BCL-2 antagonist^27^ and quinacrine and cytarabin^28^.

These studies have tended to focus on adult AML, while the paediatric disease has received relatively little attention. This study, therefore, set out to develop a rapid, high-throughput drug combination screening approach to aid in the identification of more personalised treatments for paediatric AML. High-throughput screening of drug combinations can be both time- and resource-intensive. We therefore implemented a two-stage in silico approach to inform the design of the combination screen. First, to select a drug library with the greatest potential, we carried out statistical analysis of the TARGET paediatric AML patient RNA-sequencing data, identifying “apoptosis” as a therapeutically targetable pathway. Next, to identify novel combinations, we generated an algorithm which detected all possible pairings of 80 apoptotic-inducing agents and placed them in groups of 10, across 160 wells. This used an optimisation method in which different subsets of parameters, comprising all pair combinations, examined in parallel, can be used^29^. As such, the goal was to determine a minimum solution which covers all possible pairs. Our approach reduced the combinatorial search space, balancing the quantity of reagents, consumables and time required against the ability to detect novel compound pairs.

Using three cell lines representing paediatric AML, MV4-11, CMK and Kasumi-1 lines, we observed some similarities in response to the same compound combination wells e.g. Well-47, 99 and 155. However, in the majority of cases, differences were observed between cell lines, indicating further a need for personalised medicine approaches in the treatment of paediatric AML^30,31^. The screening technique revealed a ‘novel’ drug pairing, a BCL-2 inhibitor (ABT-737) combined with a CDK2 inhibitor (Purvalanol A), with therapeutic potential in the MV4-11 cell line with a FLT3-ITD and MLLr. Unfortunately, in the FLT3-ITD AML population MLLr are rare, with only 2% of patients previously presenting with a combination of both^32^. To our knowledge, this particular drug pair has not been reported previously, however, this was not a novel mechanistic pairing as similar have been investigated by several other groups^33–35^. For example, Xie, et al.^36^ investigated LS-007, a CDK inhibitor, in combination with venetoclax against AML and observed a marked increase in apoptosis across a panel of AML cell lines. Additionally, several groups^37,38^ have investigated the inhibition of CDK9 as a way of targeting AML blasts observing down-regulation of survival genes, including MCL1. Furthermore, Venetoclax, another BCL-2 inhibitor, has recently been approved for the treatment of AML as a single agent^18^ and trials are on-going investigating combinations with either FLT3 inhibitors or demethylating agents, to test its efficacy in the treatment of AML^39,40^. These studies are a validation of the strength of the utility of our combination screening approach.

## Conclusion

Our approach has the potential to reduce resource-intensity and time in the identification of novel combination therapies. Other groups working on similar mechanistic pairings validates the potential of our approach to identify functional small molecule combinations with a larger cohort of drugs and the use of patient material.

## Materials and methods

### Differential expression and functional enrichment analyses

**Figure 7:**
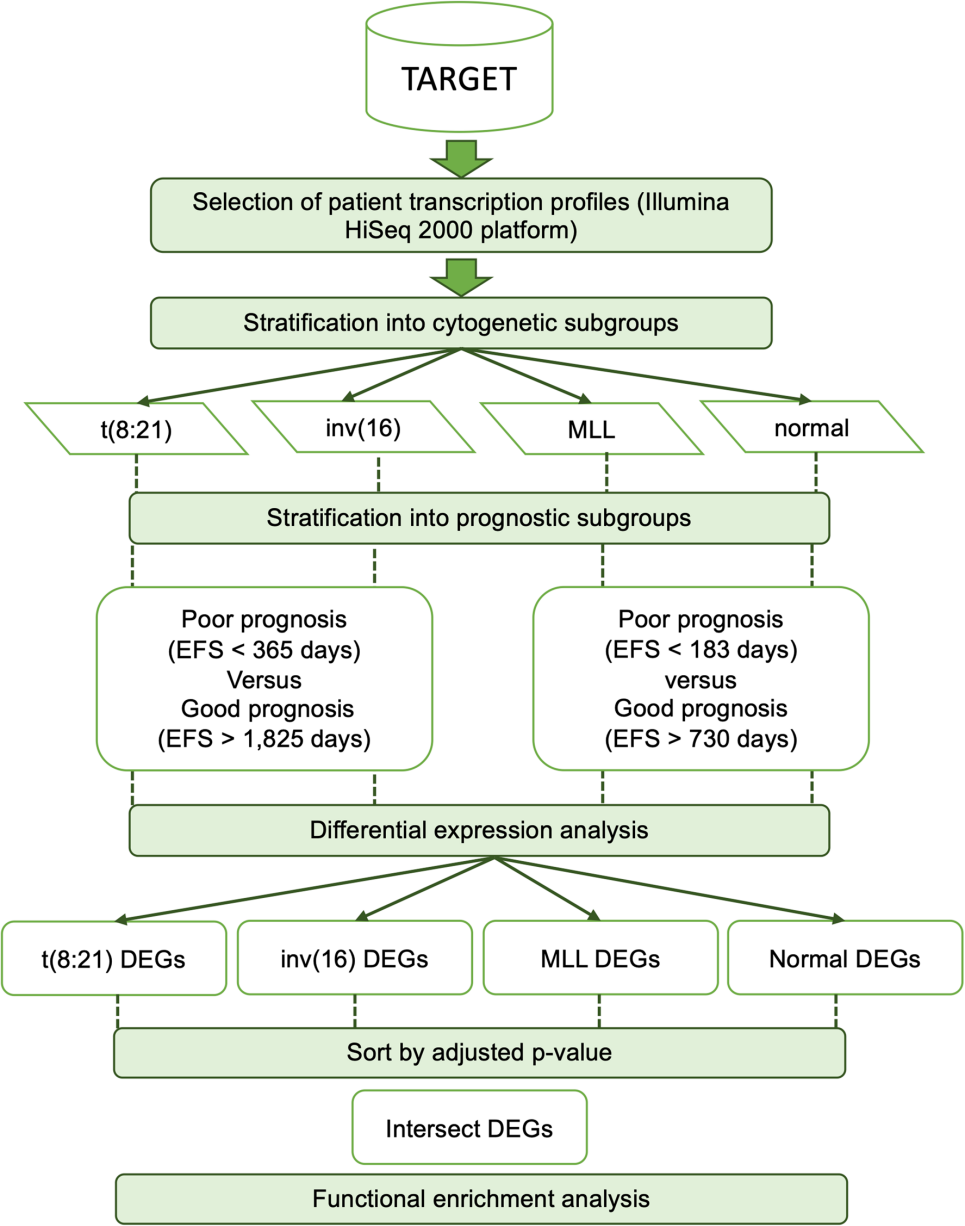
Schema of in silico analytical pipeline.

RNA-sequencing and matched clinical data from paediatric AML patients was obtained from The National Cancer Institute’s (NCI) Therapeutically Applicable Research to Generate Effective Treatments (TARGET) data matrix (https://ocg.cancer.gov/programs/target/data-matrix) and differential gene expression analysis performed. Only patients with mRNA-sequencing data obtained using the Illumina HiSeq 2000 platform were included. DESeq2, the Bioconductor package for R, was used to detect differentially-expressed genes (DEGs) using the raw count data from patients within each of the four subdivisions; t(8;21), inv(16), MLL and normal cytogenetics. Using event free survival (EFS), which is the time from study entry until an adverse event (induction failure, death, death without remission, or relapse) as defined by Bolouri et al.^5^, Kaplan–Meier curves within each cytogenetic group were plotted using the ‘survival’ package within R. Thresholds which could separate patients within each cytogenetic group into poor and good prognosis using EFS were next selected. Different selection criteria were used due to the cytogenetic risk groups affecting patient outcome. For inv(16) and t(8;21) cytogenetic groups, poor prognosis patients were defined as those with an EFS of less than one year (365 days), good prognosis was defined as an EFS of greater than five years (1825 days). For patients possessing MLL rearrangements and normal cytogenetics, EFS thresholds of 6 months (183 days) and 2 years (730 days) were used to define poor and good prognosis respectively. Prior to the DEG analysis, to remove any potential confounding, each pair of poor and good prognostic groups were balanced by gender, race, ethnicity, age at diagnosis and treatment protocol, using the R package ‘Matchlt’^41^. To obtain DEGs, data comparisons were made within each cytogenetic group between patients with poor and good EFS. Unnormalised raw count data was normalised by size factors and results were ordered by increasing adjusted p-value. Next, genes with adjusted p-values less than 0.05 were selected for Ingenuity Pathway Analysis (IPA) (QIAGEN Inc., https://www.qiagenbioinformatics.com/products/ingenuitypathway-analysis) was used to identify targetable pathways based on functional enrichment analysis of the differentially expressed genes determined within each cytogenetic risk group (normal cytogenetics, MLL, Inv(16) and t(8;21)) and the significant (p < 0.05) pathway common to the four groups was investigated. A summary chart of data analysis is shown in Fig. 7.

### Paediatric acute myeloid leukaemia cell lines

MV4-11, CMK and Kasumi-1 cell lines were used during this study to represent the clonality of AML in paediatric patients. The MV4-11 cell line was derived from a 10-year-old boy with the French-American-British (FAB) M5 subtype of AML (acute monocytic leukemia) at diagnosis^42,43^. The CMK cells were derived from a 10-month-old boy with Down's syndrome and Acute megakaryoblastic leukaemia (AML FAB M7 subtype) at relapse^44,45^. The Kasumi-1 cell line was derived from a 7-year-old boy with AML FAB M2 (acute myeloblastic leukaemia with maturation) in second relapse after bone marrow transplantation^46^. The MOLM-13, THP-1, PL-21 and CMS cell lines were used for validation experiments, each representing a case of AML with different cytogenetic and mutational backgrounds. All cell lines were obtained from the Deutsche Sammlung von Mikroorganismen und Zellkulturen (DSMZ) (Germany) except the CMS line which was provided courtesy of Dr Yubin Ge (Detroit, USA)^47^.

Each cell line was maintained in Roswell Park Memorial Institute (RPMI) 1640 (Thermo Fisher Scientific, UK) supplemented with, either 10% Fetal Bovine Serum (FBS; Thermo Fisher Scientific, UK) (MV4-11) or 20% FBS (CMK and Kasumi-1 cell lines) and 100 µg/mL Penicillin–Streptomycin (Thermo Fisher Scientific, UK). All cell lines were cultured in a humidified incubator at 37 °C supplemented with 5% carbon dioxide.

### Combination screen algorithm

Using the principle of all-pairs testing, as used in computer science^48^, an algorithm was designed for application to the multiplex screening context. Given a compound reference library of a defined size (*N*) and a set number of compounds (*n*) to be used in each plate well, the algorithm was designed to include all possible pairwise compound combinations, using the least number of wells.

Exact solutions are possible for smaller values of *N*, however as the size of the compound library increases, a compromise between computational resources and near-optimal solutions must be sought^49^. The algorithm was therefore designed to dissect the problem into stages with a local optimum solution identified at each stage and repeated occurrences of compounds pairs tolerated. Given a total of *N* compounds, *N* × (*N* − 1)/2 pairs are possible. Likewise, if *n* compounds are to be used in each well *n* ×  (*n* − 1)/2 pairs will be achieved.

With *m* wells, the problem can be redefined as minimising *m* × *n* ×  (*n* − 1)/2 so that all possible *N* ×  (*N* − 1)/2 pairs are represented. The algorithm considers a full list of compound pairs together with an active list of pairs not yet allocated to a well. At the beginning, both lists are identical. Initial random penalties are allocated to each compound pair. From the full pair list a set of ***n*** compound pairs are selected at random and their combined penalty scores evaluated until a set is obtained with a minimised (local or close to minimum) penalty score. These selected pairs are removed from the active list and the penalty scores increased (full list). This guides the next step of the search process away from previously used pairs. The algorithm stops when all possible pairs (active list) are allocated. Due to the random allocation of initial penalties, in this study the process was restarted 50 times, to determine an overall near-optimal solution. For more information see Supplementary Methods 1.

The algorithm was implemented in the R statistical programming language (https://cran.r-project.org/). Code and an accompanying tutorial are available at https://sourceforge.net/projects/geca/files/musicAL/.

### Compound screening

The Selleckchem Apoptosis compound library was used as the basis for the multiplex screen (https://www.selleckchem.com/screening/apoptosis-library.html). Compound screening assays were carried out on 96-well black optical bottom plates (Nunc, Science Warehouse Limited). Cell lines (MV4-11, CMK and Kasumi-1 cell lines) were seeded at a density of 4.0 × 10^4^ cells per well. Compounds were diluted with Dimethyl Sulfoxide (DMSO) (Sigma-Aldrich, UK) and added to cell culture to give final concentrations of 1 µM, 0.1 µM and 0.01 µM for the single agent screen or 0.1 µM for the combination screen. Cells treated with 0.1% DMSO were used as a vehicle control. The plates were then transferred to a humidified incubator at 37 °C supplemented with 5% CO_2_ for up to a 72 h incubation. Cell viability was assessed at several time-points, 24 h, 48 h and 72 h, with CellTox Green Cytotoxicity assay (Promega, UK), added to the cell culture at seeding. Relative fluorescence unit (RFU) (Ex: 485 nm, Em: 520 nm) was measured using a Synergy HTX Multi-Mode Microplate reader (Biotek, Vermont, USA) to determine cytotoxicity proportional to loss of cell membrane integrity and increased fluorescence. Unless stated otherwise, all assessment of cell viability was performed using CellTox Green Cytotoxicity assay.

The RFU values produced by the single agents in each effective well were plotted alongside the RFU value generated by the same ten compounds in combination. Successful wells, for the combination screen, were considered those with RFU values greater than three standard deviations (dotted line) from the mean (solid line) of all the data points generated from the 72 h time point per cell line. This is a common method used for identifying active compounds when performing large scale compound and RNA interference screens^50–53^.

In specific wells of interest, in order to identify potential synergistic pairs, the ten compounds were deconvolved into 45 pairwise combinations used at 0.1 µM. A full list of the compounds in each of the 160 wells is available in Supplementary Table 1. Synergistic pairs were those producing an effect greater when combined than the sum of their individual effects. In terms of combinational indices a value < 1 indicates a synergistic combination, a value of 1 indicates an additive combination and a value > 1 is indicative of an antagonistic combination^54^.

Target combinations were next tested in a wider panel of paediatric AML cell lines (MV4-11, MOLM-13, THP-1, PL-21, Kasumi-1, CMS and CMK). In brief cells were seeded at 2 × 10^5^ cells per mL of complete media, supplemented with a 1/500 dilution of CellTox Green reagent, and 100 µL added to the wells of a 96 well black optical bottom plate. Alongside each pairwise combination, the single agents were also tested to ensure an effect observed was not due to the action of a single agent. Plates were incubated in a humidified incubator at 37 °C supplemented with 5% CO_2_ for up to 72 h. Readout of cell viability was achieved using the Synergy HTX Multi-Mode Microplate reader (Biotek, Vermont, USA) to detect fluorescence (RFU values) as above at 24, 48 and 72 h post treatment.

## Supplementary information

Supplementary Information 1.

Supplementary Legends.

Supplementary Methods 1.

Supplementary Table 1.

Supplementary Table 2.

Supplementary Table 3.

Supplementary Table 4.

Supplementary Table 5.

Supplementary Table 6.
