# Supplementary material for: A compound combination screening approach with potential to identify new treatment options for paediatric acute myeloid leukaemia
Source: Sci Rep. 2020 Oct 28;10:18514. doi: 10.1038/s41598-020-75453-3 (PMC7595190; doi:10.1038/s41598-020-75453-3)
Supplement: Supplementary file 1 — Supplementary Information 1. [file 41598_2020_75453_MOESM1_ESM.pdf]

A Compound Combination Screening Approach with Potential to Identify New Treatment Options for Paediatric Acute Myeloid Leukaemia

Katrina Lappin[1*], Lindsay Davis[1], Kyle B. Matchett[1,2], Yubin Ge[3], Ken I Mills[1ǂ], Jaine Blayney[1ǂ]

1 Centre for Cancer Research and Cell Biology, 97 Lisburn Road, Queen's University Belfast, BT9 7AE

2 Northern Ireland Centre for Stratified Medicine, School of Biomedical Sciences, Ulster University, C-TRIC, Altnagelvin Hospital Campus, BT47 6SB, Derry/Londonderry, United Kingdom

3 School of Medicine, Wayne State University, 421 E. Canfield Street, Suite 3128, Detroit, MI 48201

ǂJoint senior authors

*Corresponding author – address all correspondence to K.Lappin@qub.ac.uk

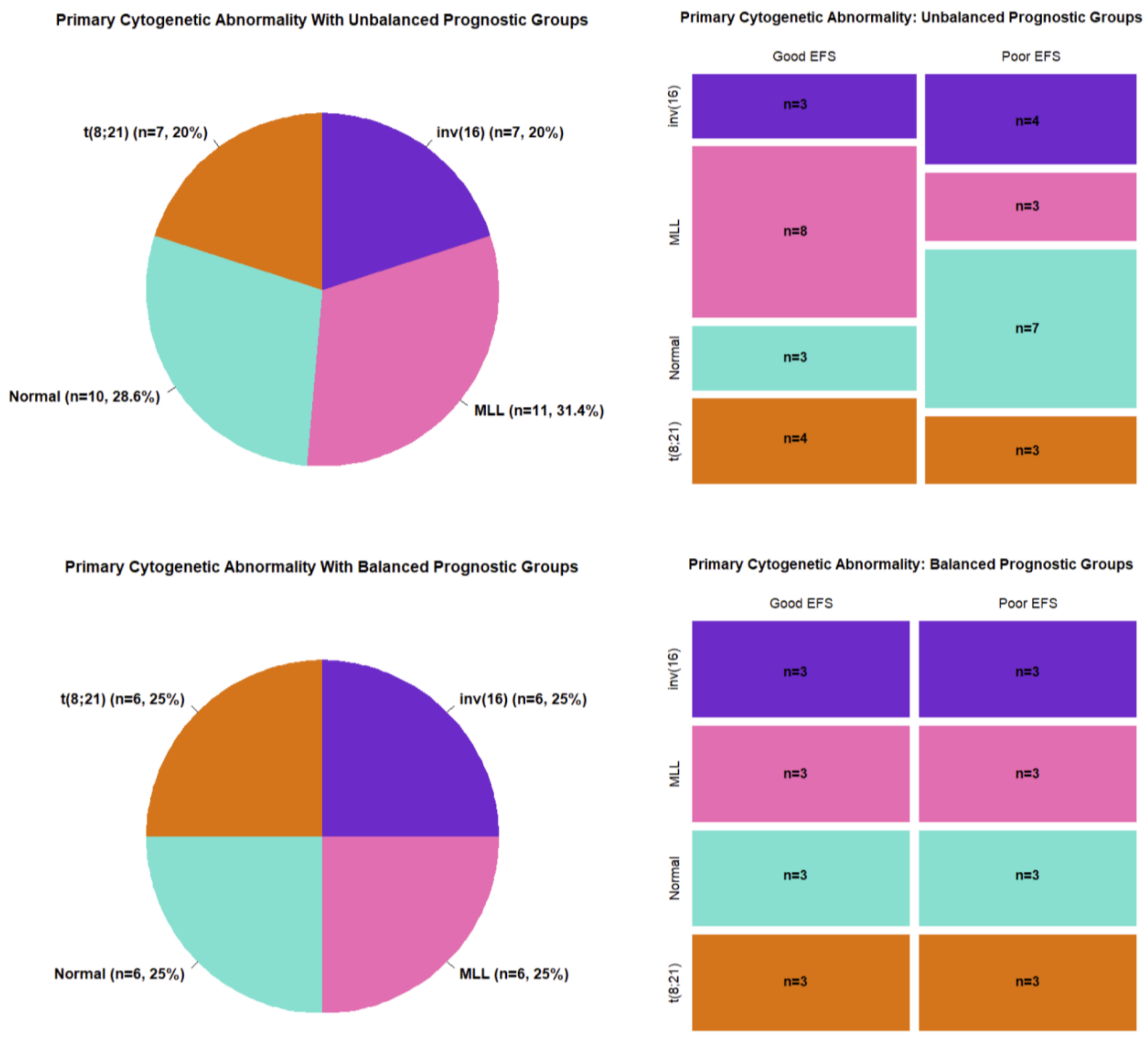

Figure S1: Balanced and unbalanced summaries for each cytogenetic group

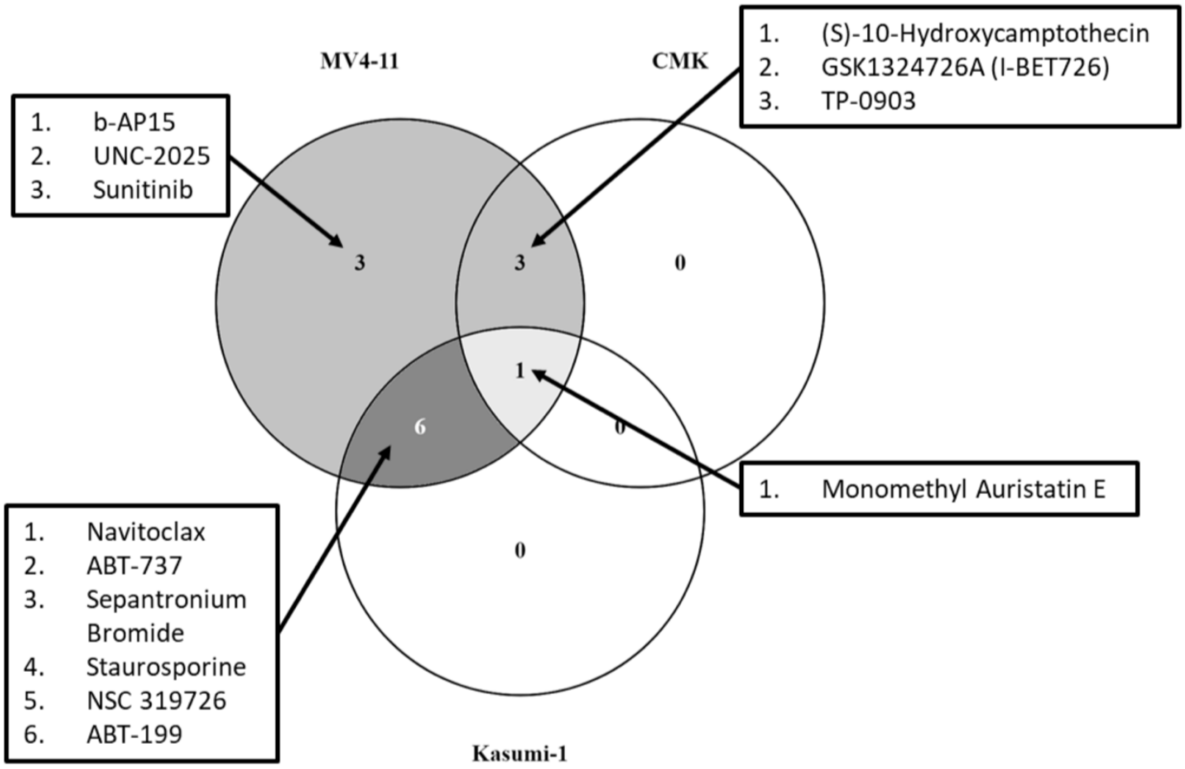

Figure S2: Venn diagram summarising the successful compounds (compounds which stimulated a relative fluorescence unit (RFU) value of greater than or equal to 2) which were common and unique across the three cell lines.

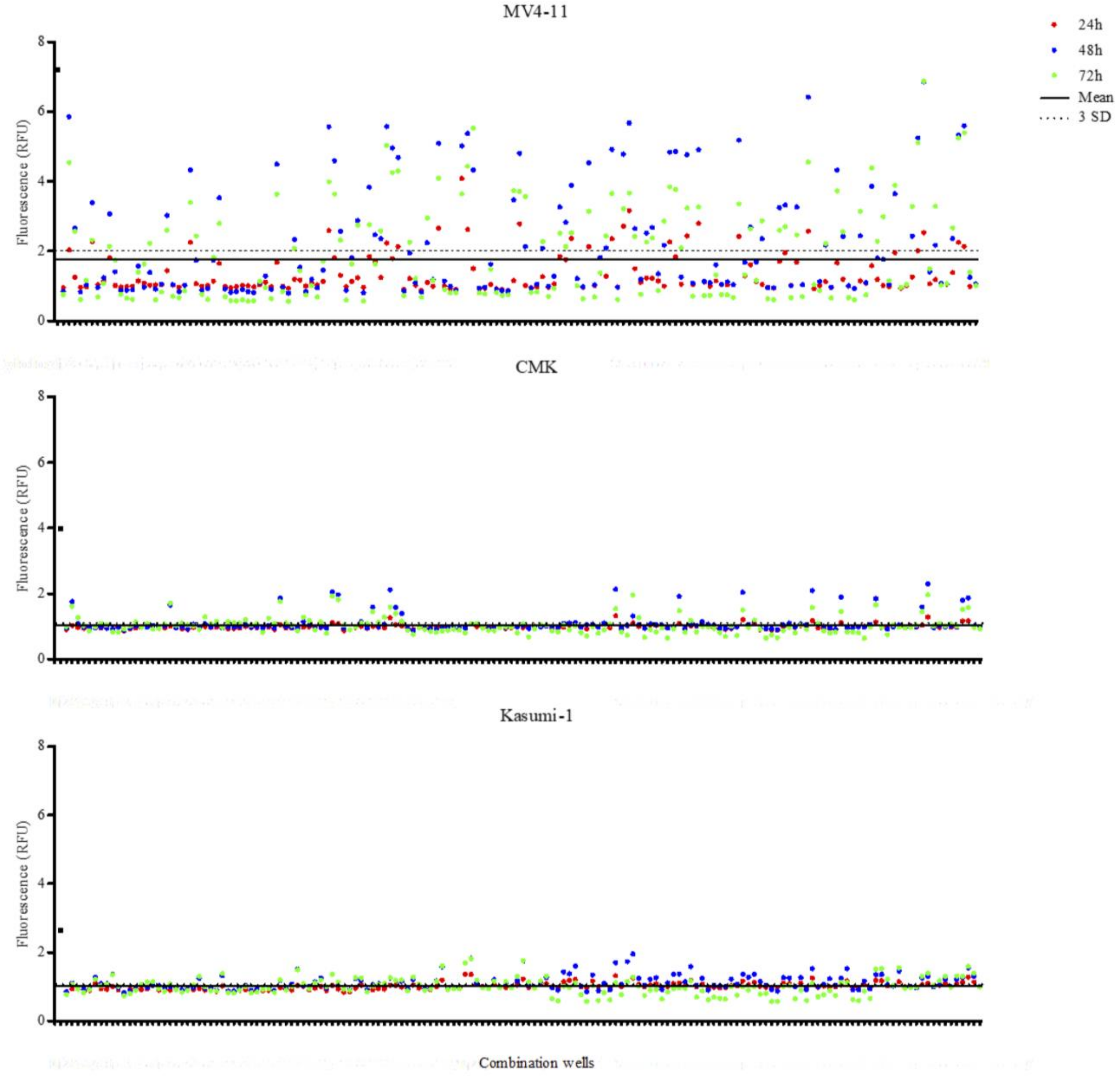

Figure S3: All data points generated from the combination screen across three time points and three cell lines

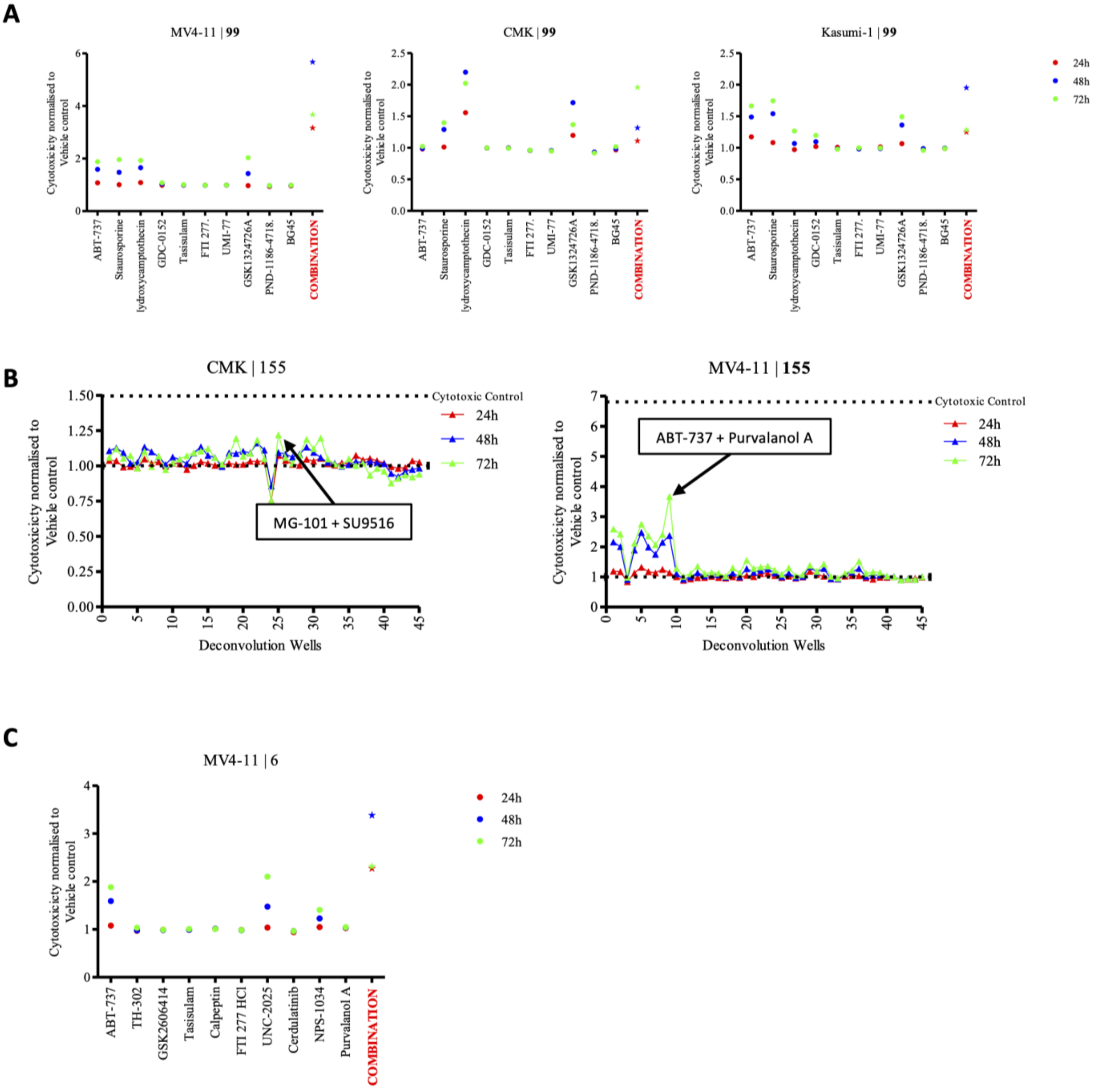

Figure S4: Data showing Well-155 was the only successful well overlapping the CMK and MV4-11 cell lines. (A) Well-99 was a common hit well (RFU >2), however analysing the RFU for the individual compounds it is clear that the effect in the combination well is an additive one of 3 or more compounds for each cell line. (B) RFU values from each time point of the deconvolution of well-155. (C) Well-6, another hit well for the MV4-11 cell line, interestingly contained the successful combination of ABT-737 and Purvalanol A identified in well-155, however the RFU value produced by the combination well is potentially an additive effect caused by ABT-737, UNC-2025 and NPS-1034.
