# Supplementary material for: A compound combination screening approach with potential to identify new treatment options for paediatric acute myeloid leukaemia
Source: Sci Rep. 2020 Oct 28;10:18514. doi: 10.1038/s41598-020-75453-3 (PMC7595190; doi:10.1038/s41598-020-75453-3)
Supplement: Supplementary file 2 — Supplementary Legends. [file 41598_2020_75453_MOESM2_ESM.docx]

**Supplementary Methods 1** Additional information regarding the combination screen algorithm

The algorithm selects each compound at random, once a compound is selected for a particular well, it is weighted in order to prohibit further selection. For this process, all compounds are treated equally, and prior knowledge of the compound activity is not required. The scale of the combinatorial problem is such that an exact solution cannot be found (global minimum), therefore an estimated solution is used (a local minimum, with some compounds repeated). The algorithm is repeated multiple times with the objective of minimising the number of wells. In minimising the number of wells used and the random aspect of the compound to well allocation, it must be accepted that there are also limitations. In undertaking this combinatorial approach, the aim is to strike a balance between the speed of discovery (minimising time and experimental resources) and the number of potential novel combinations identified. By using the alternative to pair-wise testing, the risk that potentially some interesting combinations may be missed must be accepted. There is also the potential that pairwise effects could be obscured or skewed by triplet combinations or antagonistic pairings. Therefore, a non-linear approach using random forests regression (not included in this manuscript) in which statistical interactions were considered to be surrogates of compound interactions. In each ten compound well, cell viability was modelled using the single compound values available from earlier in the experiment. Both backwards and forwards selection approaches were used to identify those combinations which had the greatest contribution towards explaining the variance, whether negative or positive. Robustness testing was also considered in which one cell line at a time was removed from the analysis. The wells in which combinations of two compounds had the greatest effect on variance were of interest, as this would potentially minimise the number of side-effects within a clinical setting. In comparing the results from the regression approach to the approach used in this manuscript, reassuringly there was considerable overlap. However, this method of analysing the output, alongside other methods e.g. Bayesian Belief Networks, requires further testing and experimental validation before we can publish. In addition, although not part of this study, the deconvolution process can be used to identify triplet or quadruplet combinations.
