# Supplementary material for: A compound combination screening approach with potential to identify new treatment options for paediatric acute myeloid leukaemia
Source: Sci Rep. 2020 Oct 28;10:18514. doi: 10.1038/s41598-020-75453-3 (PMC7595190; doi:10.1038/s41598-020-75453-3)
Supplement: Supplementary file 3 — Supplementary Methods 1. [file 41598_2020_75453_MOESM3_ESM.docx]

**Supplementary Figure legends**

**Figure S1:** Balanced and unbalanced summaries for each cytogenetic group

**Figure S2:** Venn diagram summarising the successful compounds (compounds which stimulated a relative fluorescence unit (RFU) value of greater than or equal to 2) which were common and unique across the three cell lines.

**Figure S3:** All data points generated from the combination screen across three time points and three cell lines

**Figure S4:** Data showing Well-155 was the only successful well overlapping the CMK and MV4-11 cell lines. (A) Well-99 was a common hit well (RFU >2), however analysing the RFU for the individual compounds it is clear that the effect in the combination well is an additive one of 3 or more compounds for each cell line. (B) RFU values from each time point of the deconvolution of well-155. (C) Well-6, another hit well for the MV4-11 cell line, interestingly contained the successful combination of ABT-737 and Purvalanol A identified in well-155, however the RFU value produced by the combination well is potentially an additive effect caused by ABT-737, UNC-2025 and NPS-1034.

**Supplementary Tables**

**Supplementary Table 1** Details of 80 compounds used in multiplex screen (Selleckchem apoptosis compound library) and multiplex screen design

**Supplementary Table 2** NORM EFS groups DEGs

**Supplementary Table 3** MLL EFS groups DEGs

**Supplementary Table 4** inv16 EFS groups DEGs

**Supplementary Table 5** t(8;21) EFS groups DEGs

**Supplementary Table 6** Normalised (to DMSO vehicle) data from the single agent screen of the 80 apoptosis inducing agents

**Supplementary Methods 1** Additional information regarding the combination screen algorithm
